# Multimodal magnetic resonance imaging of youth sport-related concussion reveals acute changes in the cerebellum, basal ganglia, and corpus callosum that resolve with recovery

**DOI:** 10.3389/fnhum.2022.976013

**Published:** 2022-10-19

**Authors:** Najratun Nayem Pinky, Chantel T. Debert, Sean P. Dukelow, Brian W. Benson, Ashley D. Harris, Keith O. Yeates, Carolyn A. Emery, Bradley G. Goodyear

**Affiliations:** ^1^Department of Biomedical Engineering, University of Calgary, Calgary, AB, Canada; ^2^Department of Clinical Neurosciences, University of Calgary, Calgary, AB, Canada; ^3^Hotchkiss Brain Institute, University of Calgary, Calgary, AB, Canada; ^4^Canadian Sport Institute Calgary, University of Calgary, Calgary, AB, Canada; ^5^Benson Concussion Institute, University of Calgary, Calgary, AB, Canada; ^6^Department of Radiology, University of Calgary, Calgary, AB, Canada; ^7^Alberta Children’s Hospital Research Institute, University of Calgary, Calgary, AB, Canada; ^8^Department of Psychology, University of Calgary, Calgary, AB, Canada; ^9^Department of Pediatrics, University of Calgary, Calgary, AB, Canada; ^10^Department of Community Health Sciences, University of Calgary, Calgary, AB, Canada; ^11^Sports Injury Prevention Research Centre, University of Calgary, Calgary, AB, Canada; ^12^Department of Psychiatry, University of Calgary, Calgary, AB, Canada; ^13^Seaman Family MR Research Centre, University of Calgary, Calgary, AB, Canada

**Keywords:** MRI, sport, concussion, mTBI, cerebellum, basal ganglia, youth, multimodal

## Abstract

Magnetic resonance imaging (MRI) can provide a number of measurements relevant to sport-related concussion (SRC) symptoms; however, most studies to date have used a single MRI modality and whole-brain exploratory analyses in attempts to localize concussion injury. This has resulted in highly variable findings across studies due to wide ranging symptomology, severity and nature of injury within studies. A multimodal MRI, symptom-guided region-of-interest (ROI) approach is likely to yield more consistent results. The functions of the cerebellum and basal ganglia transcend many common concussion symptoms, and thus these regions, plus the white matter tracts that connect or project from them, constitute plausible ROIs for MRI analysis. We performed diffusion tensor imaging (DTI), resting-state functional MRI, quantitative susceptibility mapping (QSM), and cerebral blood flow (CBF) imaging using arterial spin labeling (ASL), in youth aged 12-18 years following SRC, with a focus on the cerebellum, basal ganglia and white matter tracts. Compared to controls similar in age, sex and sport (*N* = 20), recent SRC youth (*N* = 29; MRI at 8 ± 3 days post injury) exhibited increased susceptibility in the cerebellum (*p* = 0.032), decreased functional connectivity between the caudate and each of the pallidum (*p* = 0.035) and thalamus (*p* = 0.021), and decreased diffusivity in the mid-posterior corpus callosum (*p* < 0.038); no changes were observed in recovered asymptomatic youth (*N* = 16; 41 ± 16 days post injury). For recent symptomatic-only SRC youth (*N* = 24), symptom severity was associated with increased susceptibility in the superior cerebellar peduncles (*p* = 0.011) and reduced activity in the cerebellum (*p* = 0.013). Fewer days between injury and MRI were associated with reduced cerebellar-parietal functional connectivity (*p* < 0.014), reduced activity of the pallidum (*p* = 0.002), increased CBF in the caudate (*p* = 0.005), and reduced diffusivity in the central corpus callosum (*p* < 0.05). Youth SRC is associated with acute cerebellar inflammation accompanied by reduced cerebellar activity and cerebellar-parietal connectivity, as well as structural changes of the middle regions of the corpus callosum accompanied by functional changes of the caudate, all of which resolve with recovery. Early MRI post-injury is important to establish objective MRI-based indicators for concussion diagnosis, recovery assessment and prediction of outcome.

## Introduction

Sport-related concussion (SRC) is a mild traumatic brain injury (mTBI) sustained during sport, induced by an impulsive biomechanical force directly to the head, face or neck, or by a transmitted force to the head from other parts of the body (McCrory et al., 2017). These forces often result in short-lived neurological symptoms (e.g., dizziness, headache, light and noise sensitivity, difficulty concentrating and remembering, fatigue, confusion, irritability and sadness) that resolve within hours or days; however, in up to 30% of youth concussion cases (12–18 years of age), neurological symptoms can persist for weeks or even months (Barlow et al., 2010, 2011; Babcock et al., 2013; Zemek et al., 2013, 2016; Schneider et al., 2021), making youth concussion a major health issue. While clinical assessments, including the SCAT5 (McCrory et al., 2017) and 5P score (Zemek et al., 2016), possess diagnostic sensitivity and help predict outcome, it cannot determine the underlying neurological mechanisms for a fast or delayed recovery. This hinders the clinical management and treatment of youth SRC, and it does not permit an estimate of when and if youth will recover to return to their sport and daily activities.

Concussion does not typically lead to visible abnormalities on structural neuroimaging. Recently, advanced magnetic resonance imaging (MRI) methods have shown promise to diagnose concussion and predict outcomes (Lunkova et al., 2021); however, the wide range in concussion symptoms, symptom severity, time of MRI post-injury and subject demographics, even within the same study, have hindered consistency across studies in terms of the brain regions impacted by concussion. One approach to better define the role of MRI in concussion is to first focus investigations on brain regions that play a role across commonly reported symptoms using MRI techniques that are sensitive to proposed neurological mechanisms. This approach could provide a better neurological understanding of prolonged concussion symptoms and could help develop more accurate models for predicting recovery trajectories.

The cerebellum plays a major role in motor and cognitive functions, and is associated with common concussion symptoms like dizziness and disorientation, yet it has received little attention to date in its role in concussion. Several studies have reported post-concussive structural abnormalities within the cerebellar peduncles, the nerve tracts that connect the cerebellum to the brainstem and cerebrum (Jang et al., 2016; Mallott et al., 2019). Structural deficits of nerve tract bundles can be detected by diffusion tensor imaging (DTI), which measures the random movement of water molecules in the vicinity of white matter tracts via a number of metrics, including: mean diffusivity (MD, the ability of water to diffuse in any direction, sometimes expressed as the apparent diffusion coefficient), axial diffusivity (AD, the ability of water to diffuse along the direction of white matter tracts), radial diffusivity (RD, the ability of water to diffuse across white matter tracts), and fractional anisotropy (FA, a scale of 0 to 1 that quantifies the directional dependence of water diffusion, where 0 indicates total isotropic movement and 1 indicates movement along a single direction). A growing number of studies have used DTI to report changes in white matter following concussion (Cubon et al., 2011; Bazarian et al., 2012; Borich et al., 2013; Virji-Babul et al., 2013; Murugavel et al., 2014; Sasaki et al., 2014; Lancaster et al., 2016; Orr et al., 2016; Churchill et al., 2018; Mustafi et al., 2018; Wu et al., 2018, 2020); however, affected white matter regions vary greatly between studies.

One animal model study of pediatric mTBI reported both acute and persistent alterations within the cerebellum that are consistent with the activation of immune cells during an inflammatory response (Fraunberger et al., 2020). In response to inflammation, iron can accumulate within inflamed tissues (Reichenbach et al., 2015). Quantitative susceptibility mapping (QSM) is an MR technique that can provide brain images of the degree of magnetic susceptibility (i.e., magnetic field distortion) caused by specific biomarkers such as iron in tissues (Wang et al., 2017). QSM has only recently been proposed as a potential biomarker for concussion (Koch et al., 2018; Brett et al., 2021), and it has not yet been used in detail to investigate white matter injury following mTBI.

The basal ganglia are also involved in a variety of motor and cognitive functions, including motor control, motor learning, executive function, behavioral regulation and emotional processing. These functions span many of the symptoms commonly reported following concussion. One functional MRI study of mTBI subjects demonstrated reduced activation of the basal ganglia and cerebellum relative to controls during a visual memory task (Yang et al., 2012), and a resting-state fMRI study found that functional connectivity of the caudate was positively correlated with mental fatigue (Lewis et al., 2018), another symptom commonly reported by concussion sufferers. Together, the cerebellum and basal ganglia have also been shown to be associated with abnormal metabolic cascade in acute TBI patients (Hattori et al., 2003). Hence, the cerebellum and basal ganglia seem plausible as region-of-interest candidates for the development of MRI-based indicators of concussion symptoms and their persistence or recovery.

In the present study, we used multimodal MRI, including DTI, QSM and resting-state fMRI to investigate youth SRC, with a focus on the cerebellum, basal ganglia and the white matter tracts that project from them, with consideration for symptom severity and time between injury and MRI. In addition, we included arterial spin labeling (ASL) MRI to investigate cerebral blood flow (CBF) following concussion, as it has been used in a number of previous studies, but with mixed findings (Len and Neary, 2010; Maugans et al., 2012; Barlow et al., 2016, 2017; Wiseman et al., 2017; Stephens et al., 2018; Wang et al., 2019, 2016, 2015). Finally, we used resting-state fMRI data to investigate changes in the level of spontaneous neural activity following concussion [via the amplitude of low-frequency fluctuations (ALFF)] (Zang et al., 2007; Zhan et al., 2016; Shi et al., 2021). We hypothesized that the cerebellum and basal ganglia exhibit acute changes in MRI measures of structure and/or function in association with symptoms after concussion and time between injury and MRI, and that these changes resolve with recovery from symptoms.

## Materials and methods

This study was approved by the University of Calgary’s Conjoint Health Research Ethics Board. Twenty-nine recently concussed youth ice hockey players between 12 and 18 years of age (15.7 ± 1.3 years; 23 male, 6 female) were recruited from the Benson Concussion Institute BRAIN cohort study (Calgary, Alberta, Canada) and the Sport Injury Prevention Research Centre Safe-to-Play cohort study (Faculty of Kinesiology, University of Calgary, Calgary, AB, Canada) to undergo MRI as soon as possible following injury. Initial symptom severity was taken as the total score on the SCAT version 3 (SCAT3) assessment when recruited into their respective studies at a median of 3 days post injury (range 1–6 days). Participants underwent MRI at a median of 8 days post injury (range 2–15 days). Sixteen youth ice hockey players who experienced a concussion and had been cleared by a physician to return to play hockey were also recruited (age: 14.8 ± 1.9 years; 14 male, 2 female). These participants were initially assessed for symptom severity at a median of 4 days post injury (range 1–8 days) and underwent MRI at 41 ± 16 days post injury, which was 11 ± 9 days following clearance to return to play (median SCAT3 score of 0 when cleared). Twenty healthy youth control participants were recruited (age: 14.5 ± 1.9 years; 16 male, 4 female), similar in age, sex and sport to the recently concussed and recovered groups. Exclusion criteria included a previous history of chronic medical conditions (e.g., metabolic or nephritic disorders) or a pre-existing neurological condition (e.g., stroke, seizure, moderate-to-severe TBI, congenital intracranial abnormalities).

MRI data were acquired using a 3 Tesla GE Discovery MR750 scanner (GE Healthcare, Waukesha, WI) at one of two sites (Seaman Family MR Research Centre and the Alberta Children’s Hospital, both located at the University of Calgary), running identical software versions. All image processing was performed using software tools within *FSL*,^1^ unless otherwise indicated. High-resolution 3D T1-weighted images were acquired in the sagittal plane for anatomical registration of MRI data using GE’s BRAVO sequence, which is an inversion recovery-prepared fast spoiled gradient echo sequence [voxel size = 1.0 mm isotropic; repetition time (TR) = 6.7 ms; echo time (TE) = 2.9 ms; flip angle = 10°; matrix = 256 × 256 × 192]. These images were skull stripped using the brain extraction tool (*BET*) of *FSL*, and were subsequently registered to the MNI152 template brain using *FSL*’s linear registration tool (*FLIRT*) (Jenkinson et al., 2002). The cerebellum and basal ganglia (including the putamen, pallidum and caudate) were then segmented from the images using *Freesurfer v6.0*.^2^ Additional regions of interest (ROIs) were segmented to investigate their functional connectivity with the cerebellum and basal ganglia, which were based on previous MRI studies demonstrating associations with mTBI. These ROIs included the thalamus, amygdala, parahippocampal gyrus, hippocampus, precuneus, insula, middle frontal gyrus, supramarginal gyrus, and inferior and superior parietal lobules (Messé et al., 2013; Stevens et al., 2012; Yang et al., 2012; Keightley et al., 2014; Iraji et al., 2015; Mckee and Daneshvar, 2015; Nathan et al., 2015; Yan et al., 2016; Murdaugh et al., 2018; Zivadinov et al., 2018; Li et al., 2019; Spader et al., 2019; Zhang et al., 2020). Given the possible involvement of these brain regions, we also examined them for changes in ALFF, CBF and susceptibility following concussion.

DTI data were collected using a spin-echo echo planar imaging sequence (fat saturation RF pulse, 32 diffusion directions, 3 T2-weighted images, 220-mm field of view, 100 × 100 matrix reconstructed at 128 × 128, 2.2-mm slice thickness, TR/TE = 7000/80 ms, b = 750 or 1000 s/mm2). Following eddy current correction using *FSL*’s *eddy* tool^3^ (Andersson and Sotiropoulos, 2016), *FSL*’s *dtifit*^4^ was used to generate whole-brain FA, MD, AD, and RD images. *FSL*’s Tract Based Spatial Statistics (*TBSS*) tool^5^ (Smith et al., 2006) was used to register FA images to the MNI152 brain template at a final resolution of 2 mm × 2 mm × 2 mm. The mean FA image across participants was created and thinned to create a mean FA skeleton, which represents the centers of all tracts common to the group. Each participant’s aligned FA data was then projected onto this skeleton to permit group-based analyses. These steps were then applied to the MD, AD, and RD maps. To remove outlier image voxels, DTI maps were concatenated across participants and temporal independent component analysis (ICA) was performed using *FSL*’s *MELODIC* tool^6^ to remove, by regression, voxel intensity profiles across participants that exhibited a spike in intensity for a single participant. White matter tract regions of interest connecting or projecting from the cerebellum and basal ganglia (internal capsule, external capsule, uncinate fasciculus, corona radiata, anterior thalamic radiation, and the superior, middle and inferior peduncles of the cerebellum) were identified using a standardized white matter atlas in *FSL* (i.e., JHU ICBM white matter labels and JHU white matter tracts), and the corpus callosum was segmented into five subregions (anterior, mid-anterior, central, mid-posterior, posterior) using *Freesurfer*. For all regions of interest, mean FA, MD, AD, and RD were calculated for each participant.

QSM data were collected using a radio frequency-spoiled, flow-compensated 3D gradient echo sequence (TR/TE = 29.5/26.3 ms; flip angle = 10°; matrix = 256 × 256 × 132; voxel size = 1 mm isotropic; 8 echoes). QSM images (i.e., magnetic susceptibility images) were then generated using *Cerebra-QSM* (Salluzzi et al., 2017). For the purposes of group analysis, voxel intensity in the susceptibility maps for each participant was converted to a Z-score relative to the whole-brain average, given that we were interested in determining if ROIs deviated from normal rather than determining absolute susceptibility differences between groups. Thus, the mean Z-score was computed for the all regions of interest, including the white matter ROIs, for group analysis.

ASL MRI was performed using a 3D stack-of-spirals pseudo-continuous ASL (pCASL) fast spin echo sequence (1.45-s label duration, post-label delay = 2.025 s, 8 spiral stacks, 30 slices, acquired resolution 3.66 mm × 3.66 mm, slice thickness = 3.5 mm, field of view = 229 mm, 3 averages) with an additional 2-s saturation recovery image to permit the calculation of CBF during post-processing. The bottom of the 3D inversion slab was positioned at the bottom of the cerebellum. Quantitative CBF maps were then calculated using vendor-provided software (GE Healthcare), and two-step image registration was performed to align the CBF maps to the T1-weighted images and subsequently to the MNI152 template. As for the QSM data, Z-score images of the CBF maps were calculated relative to the whole-brain average, and the average Z-score was computed for all brain regions for group analysis.

Resting-state fMRI images were collected using a gradient-echo echo-planar image sequence (TR/TE = 2,000/30 ms, flip angle = 70°, FOV = 23 cm, matrix size = 64 × 64, slice thickness = 3.6 mm, 150 volumes). Participants were instructed to keep their eyes open and focused on a fixation cross, which was projected onto a screen positioned at the back of the MR scanner bore and viewed using an angled mirror positioned above the eyes. Processing of the fMRI data using *FSL* included scalp removal, motion correction (*MCFLIRT*), slice timing correction of the interleaved slice acquisition, spatial smoothing (full-width half-maximum = 6 mm) and high-pass temporal filtering (>0.01 Hz). A two-step registration was performed (fMRI to T1-weighted to MNI152 template) using a linear normal search (12 degrees of freedom), resulting in images at a resampled resolution of 3 mm. Independent component analysis (ICA) was performed on each participant’s data, and those components considered as artifact were removed by regression (Kelly et al., 2010; Christov-Moore, 2013). The fMRI data were then used to extract the average timeseries of all voxels within each of the cerebellum, caudate, pallidum and putamen of each hemisphere separately, plus all other ROIs. For all possible pairs of ROIs, functional connectivity was computed as the Pearson correlation coefficient (*r*) of the temporal cross-correlation of the ROI pair. These coefficients were then subject to a Fisher transformation to create a normal Z distribution for analysis. In addition, the fMRI data were also used to calculate ALFF, the sum of signal power over the 0.01-0.1 Hz frequency band, using the *DPABI/DPARSF* toolbox.^7^ As for the QSM and CBF data, ALFF was converted to Z-score relative to the whole-brain average. The mean Z-score was then computed for the cerebellum, caudate, putamen and pallidum for group analysis. Mean Z-scores for each ROI were analyzed.

All statistical analyses were performed using IBM SPSS Statistics for Macintosh, Version 27.0 (IBM Corp. Armonk, NY, USA. Release10d 2020). Age was compared between groups by one-way analysis of variance. Initial total SCAT3 severity scores were first examined for normality using the Shapiro-Wilk test and were found to not be normally distributed (W = 0.90, df = 24, *p* = 0.03). Thus, initial SCAT3 scores were log_10_ transformed to generate a normal distribution, as confirmed by an additional Shapiro-Wilk test (W = 0.97, df = 24, *p* = 0.60). Initial SCAT3 severity scores were then compared between the recent and recovered groups by a Student’s *t*-test.

ALFF, CBF, susceptibility, FA, MD, AD, and RD were separately analyzed using analyses of covariance (ANCOVAs), with group, sex and MR scanner as between-subject factors (as well as b-value for DTI metrics), including the interaction between sex and group. Hemisphere was a within-subject factor (with the exception of the corpus callosum) and age was included as a covariate. For functional connectivity analysis, connectivity of ROI pairs was entered into an ANCOVA, with group, sex and MR scanner as between-subject factors, including the interaction between sex and group. Brain region and hemisphere were within-subject factors, and age was included as a covariate. For example, for analysis of connectivity between the cerebellum and thalamus, the within-subject factors were hemisphere (left, right) and brain region (cerebellum, thalamus). For factors exhibiting a significant effect of group, pairwise comparisons were performed and corrected using the Sidak correction method. For all analyses, an alpha value of 0.05 was considered as statistically significant, and Cohen’s *d* was calculated to determine effect sizes.

Analyses were then performed on participants of the recent SRC group, to determine any associations between MRI and each of symptom severity score and number of days between injury and MRI. First, symptom severity score, number of days between injury and MRI, and age, were examined for collinearity using Pearson’s correlation analysis to ensure they were independent covariates. Five recently concussed youth had an initial SCAT3 score of 2 or less, well below the scores of the remainder of the group. These participants were excluded from the within-group analysis of symptom severity as they formed a low symptom severity score cluster separate from the remainder of participants, and would thus bias correlation analyses towards statistical significance. This left 24 symptomatic recently concussed youth for analysis. Because mTBI symptoms have been shown to improve or resolve rapidly, even partially, over the first few days following injury (McCrory et al., 2017), the number of days post-injury were log_10_ transformed to approximate a more linear association with the dependent variables under investigation. ALFF, CBF, susceptibility, FA, MD, AD, and RD were each averaged across hemispheres (given that there were no effects of hemisphere for any analysis) and analyzed by ANCOVA, with sex and MR scanner as between-subject factors (as well as b-value for DTI metrics), and initial SCAT3 symptom severity score, age, and days between injury and MRI as covariates. Functional connectivity for each ROI pair was averaged across hemispheres and also analyzed using an ANCOVA. When a significant association with symptom severity or day of MRI was found, the data were plotted as Z-scores with respect to the control group to ensure that the data weren’t centered about Z = 0, which would indicate a distribution that was the same as controls and happened by chance to be significantly associated with symptom severity or day of MRI. For significant associations with symptom severity, an ANCOVA analysis was used to determine if the five recently concussed asymptomatic youth excluded from analysis significantly differed from control participants. For all analyses, an alpha value of 0.05 was considered as statistically significant, and effect size was calculated as the *R*-squared value from Pearson’s correlation analysis.

## Results

### Subject demographics

Subject demographics are summarized in Table 1. Age did not differ between groups [*F*(2,64) = 2.25, *p* = 0.11]. Initial SCAT3 total symptom severity score did not significantly differ between recently concussed and recovered youth [*t*(43) = 0.11, *p* = 0.92], confirming that both groups were equivalent in terms of initial concussion severity. For symptomatic recently concussed youth, there were no significant correlations between age, initial symptom severity, and number of days between injury and MRI. Thus, neither age nor symptom severity were associated with the number of days between injury and MRI. Three recently concussed youth and one recovered youth had a known history of migraine. One recently concussed youth, two recovered youth, and one healthy control youth had a previous diagnosis of ADHD. Approximately 72% of recently concussed youth and 50% of recovered youth reported a previous concussion. Due to low subject numbers within these subgroups, no analyses were performed in association with migraine history, ADHD diagnosis or previous concussion.

**TABLE 1:** Summary of subject demographics. Groups did not significantly differ by age and initial symptom severity score.

	Recently concussed (*N* = 29)	Recovered (*N* = 16)	Healthy controls (*N* = 20)
Males/females	23/6	14/2	16/4
Age in years (range)	15.7 ± 1.3 (12–18)	14.8 ± 1.9 (12–18)	14.5 ± 1.9 (12–18)
Time between Injury and MRI, days	8 ± 3	41 ± 16	N/A
Total initial symptom severity score, SCAT3	23.2 ± 19.6	23.8 ± 17.9	N/A
History of Migraine, *N*	3	1	0
ADHD diagnosis, *N*	1	2	2
Self-reported previous concussion(s), *N*	21	8	1

### Between-group differences

**FIGURE 1:**
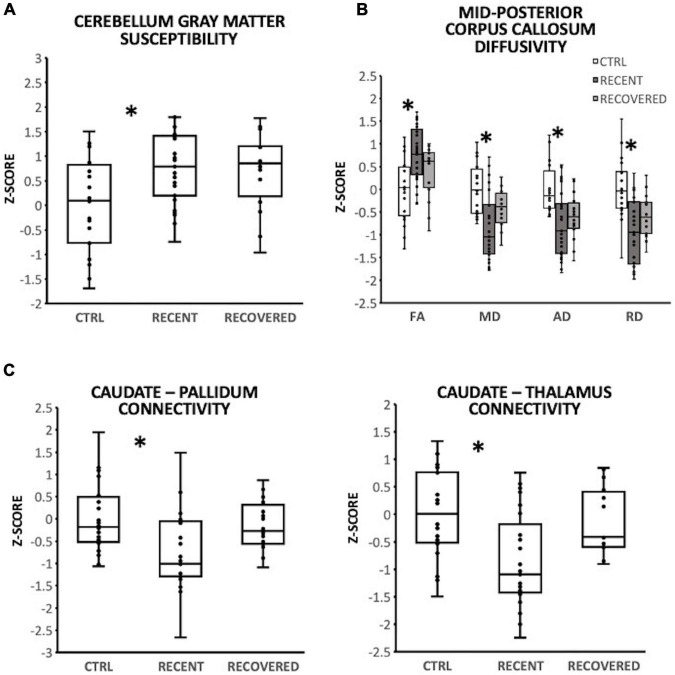
Group differencesin magnetic resonance imaging (MRI), with individual data plotted as a Z-score relative to the mean of control participants (CTRL; *N* = 20). *Indicate a significant difference (*p* < 0.05) between recently concussed youth and control participants. Relative to control participants (CTRL, *N* = 20), recently concussed youth (RECENT, *N* = 29) exhibited: **(A)** significantly increased susceptibility in the cerebellum gray matter (*p* = 0.032); **(B)** significantly increased FA (*p* = 0.038) and reduced MD (*p* = 0.017), AD (*p* = 0.022), and RD (*p* = 0.007) in the mid-posterior corpus callosum; and **(C)** significantly decreased functional connectivity between the caudate and each of the pallidum (*p* = 0.035) and thalamus (*p* = 0.021). Youth recovered from SRC (RECOVERED; *N* = 16) exhibited no significant differences from control participants.

In the cerebellum gray matter, there was a significant group effect for susceptibility [*F*(2,57) = 3.64; *p* = 0.032]. Pairwise comparisons demonstrated that relative to controls, susceptibility was significantly greater for recently concussed youth (*p* = 0.044, Cohen’s *d* = 0.73), but not for recovered youth (Figure 1A). A significant effect of group was observed in the mid-posterior region of the corpus callosum (CC) for FA [*F*(2,56) = 3.47; *p* = 0.038], MD [F(2,56) = 4.38; *p* = 0.017], AD [F(2,56) = 4.29; *p* = 0.018] and RD [*F*(2,56) = 5.39; *p* = 0.007]. Pairwise comparisons demonstrated that relative to controls, the recently concussed group exhibited significantly greater FA (*p* = 0.033, Cohen’s *d* = 0.82) and significantly lower MD (*p* = 0.014; Cohen’s *d* = 0.92), AD (*p* = 0.022, Cohen’s *d* = 0.75) and RD (*p* = 0.007, Cohen’s *d* = 1.00); the recovered group did not differ from the control group (Figure 1B). There was also a significant group effect for functional connectivity between the caudate and each of the pallidum [*F*(2,57) = 3.57; *p* = 0.035] and thalamus [*F*(2,57) = 4.13; *p* = 0.021]. Pairwise comparisons revealed that relative to controls, functional connectivity was significantly lower in recently concussed youth (caudate-pallidum: *p* = 0.036, Cohen’s *d* = 0.76; caudate-thalamus: *p* = 0.023, Cohen’s *d* = 0.81), but not recovered youth (Figure 1C).

### Symptomatic recently concussed

**FIGURE 2:**
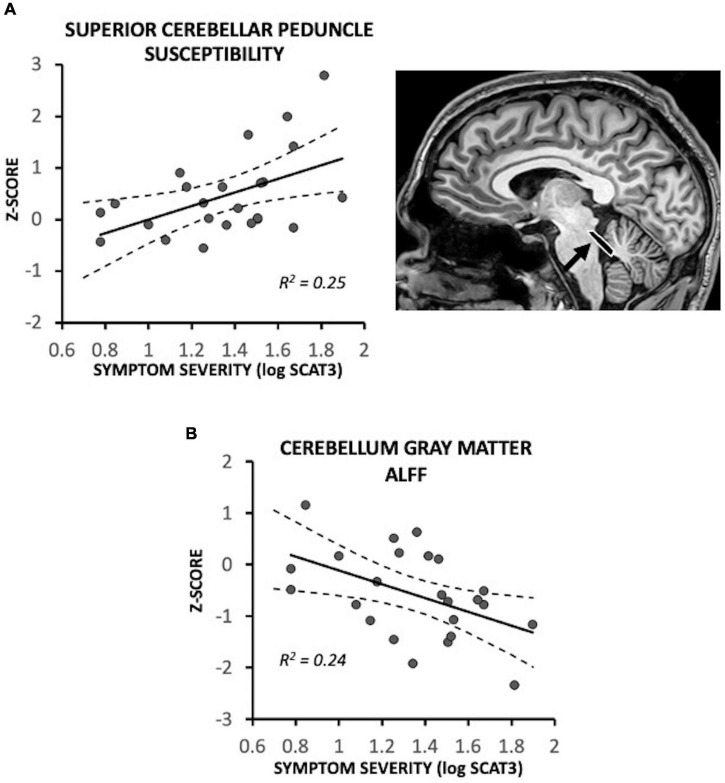
Magnetic resonance imaging (MRI) measures associated with symptom severity in symptomatic recently concussed youth (*N* = 24), expressed as a Z-score relative to control participants (*N* = 20). Greater symptom severity was significantly associated with **(A)** increased susceptibility in the superior cerebellar peduncles (*p* = 0.011) and **(B)** decreased ALFF of the cerebellum (*p* = 0.013). Low symptom severity was associated with near control participant levels (i.e., Z = 0).

In recently concussed symptomatic participants, the superior cerebellar peduncles exhibited a positive association between susceptibility and initial symptom severity [*F*(1,18) = 8.00, *p* = 0.011; *R*^2^ = 0.25]; participants with low initial symptom severity exhibited near control participant levels (i.e., Z = 0) (Figure 2A). Recently concussed asymptomatic participants did not differ from control participants [*F*(1,19) = 1.28, *p* = 0.27]. Greater symptom severity was also associated with lower ALFF in the cerebellum gray matter [*F*(1,18) = 7.59, *p* = 0.013; *R*^2^ = 0.24] (Figure 2B); participants with lower severity scores were near control participant levels (Z = 0). Asymptomatic recently concussed participants did not differ from controls [*F*(1,19) = 3.95, *p* = 0.062].

**FIGURE 3:**
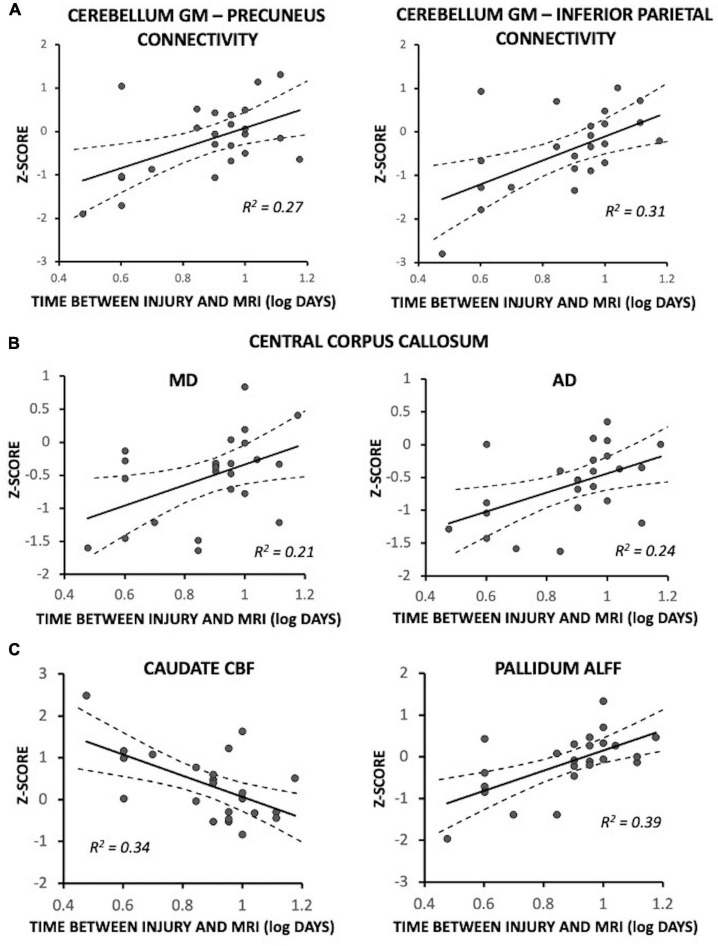
Magnetic resonance imaging (MRI) measures associated with time between injury and MRI in symptomatic recently concussed youth (*N* = 24), expressed as a Z-score relative to control participants (*N* = 20). Fewer days between injury and MRI was significantly associated with: **(A)** decreased functional connectivity between the cerebellum and each of the precuneus (*p* = 0.014) and the inferior parietal lobule (*p* = 0.007); **(B)** decreased MD (*p* = 0.037) and AD (*p* = 0.022) of the central corpus callosum; and **(C)** increased CBF in the caudate (*p* = 0.005) and decreased ALFF of the pallidum (*p* = 0.002). More days between injury and MRI was associated with near control participant levels (i.e., Z = 0).

Fewer days between injury and MRI was significantly associated with reduced functional connectivity between the cerebellum gray matter and each of the precuneus [*F*(1,18) = 7.47, *p* = 0.014; *R*^2^ = 0.27] and inferior parietal lobule [*F*(1,18) = 9.22, *p* = 0.007; *R*^2^ = 0.31] (Figure 3A); participants who had MRI at later timepoints exhibited connectivity near control participant levels. The five recently concussed asymptomatic participants did not differ from control participants for cerebellum-precuneus connectivity [*F*(1,19) = 1.96, *p* = 0.18] or cerebellum-inferior parietal lobule connectivity [*F*(1,19) = 2.80, *p* = 0.11]. In the central corpus callosum, fewer days between injury and MRI was significantly associated with lower MD [*F*(1,17) = 5.10, *p* = 0.037; *R*^2^ = 0.21] and AD [*F*(1,17) = 4.50, *p* = 0.049; *R*^2^ = 0.24]; MA and AD were near control participant levels (i.e., Z = 0) at later timepoints (Figure 3B). The five recently concussed asymptomatic participants did not differ from control participants for MD [*F*(1,19) = 0.055, *p* = 0.82] or AD [Z = −0.13 ± 0.19; *F*(1,19) = 0.026, *p* = 0.87]. Fewer days between injury and MRI was also significantly associated with greater CBF in the caudate [*F*(1,18) = 10.42; *p* = 0.005; *R*^2^ = 0.34] (Figure 3A) and lower ALFF of the pallidum [*F*(1,18) = 12.22; *p* = 0.002; *R*^2^ = 0.39] (Figure 3C); participants at later days were near control participant level (Z = 0). The five asymptomatic recently concussed participants did not differ from control participants for CBF of the caudate [*F*(1,19) = 0.95, *p* = 0.34] or ALFF of the pallidum [*F*(1,19) = 1.47, *p* = 0.24].

## Discussion

We have demonstrated using multimodal MRI that youth SRC is associated with acute physiological changes in the cerebellum, basal ganglia (primarily the caudate), and the mid-to-posterior portions of the corpus callosum. These acute changes result from inter-related mechanisms of injury, which single modality MRI would not be able to demonstrate, and these changes all resolve with the recovery of symptoms and over time.

Quantitative susceptibility mapping revealed that magnetic susceptibility is increased in the cerebellum gray matter following youth SRC, which we interpret as an acute inflammatory response, given it has been shown that an increase in susceptibility indicates the accumulation of iron that accompanies an inflammatory response (Urrutia et al., 2021). An increase in iron has been observed in mTBI studies previously (Raz et al., 2011; Lu et al., 2015), but our study is the first to report an increase in iron within the cerebellum gray matter following youth SRC. This is in agreement with a previous study that reported activation of immune cells and a cerebellar inflammatory response after pediatric mTBI in a rat model (Fraunberger et al., 2020). We also showed that this increase in cerebellum gray matter susceptibility was accompanied by a symptom severity dependent decrease in neural activity of the cerebellum gray matter, as inferred from ALFF. Given that inflammation can lead to a cellular energy crisis (Sankowski et al., 2015), resulting in inhibition of spontaneous regional activity, our results could suggest that head impact causes an inflammatory response within the cerebellum, which leads to a decrease in cerebellar activity and subsequently manifests as concussion symptoms; however, causality cannot be confirmed in the present study.

We found further evidence of a cerebellar inflammatory response in the form of a symptom severity dependent increase in magnetic susceptibility within the superior cerebellar peduncles, which connect the cerebellum to the brainstem. Further to that, we found that functional connectivity between the cerebellum and the precuneus and inferior parietal lobule was dependent on the day when MRI was performed. While not directly associated with symptom severity, the resolution of cerebellar connectivity over time in tandem with a reduction in cerebellar inflammation and an increase in cerebellar activity as symptoms resolve, suggests that cerebellar connectivity may be associated with additional symptoms not assessed by the SCAT, symptoms that are subclinical, or a symptom severity scale that differs from a self-reported scale. The latter could further suggest that changes on MRI may be a more objective indicator of injury than those inferred from self-reported symptoms.

We did not observe any changes in diffusivity within the cerebellum that could indicate axonal injury or demyelination. One previous study (Mallott et al., 2019) reported a decrease in AD in the cerebellar peduncles following concussion, whereas we observed an inflammatory response. The accumulation of iron in the vicinity of a white matter tract could impede the diffusion of water along the tract and hence decrease AD without axonal injury. It is possible that a change in AD or any other measure of diffusivity may have been too small to detect in our study population.

We observed a reduction in water diffusivity in the mid-posterior region of the corpus callosum as well as an acute day-dependent reduction in water diffusivity in the central corpus callosum, possibly indicating axonal injury that resolved over time. The middle portions of the corpus callosum consist of between-hemisphere connections of the frontoparietal, somatomotor and ventral attention networks (Wang et al., 2020; Liu et al., 2021), which play roles in modulating cognitive control, enabling motor and sensory based activities, and regulating involuntary actions (Zanto and Gazzaley, 2013). It is thus plausible that acute changes within the mid-to-posterior regions of the corpus callosum are associated with cognitive, attention, concentration, motor and sensory symptoms commonly reported in the SCAT following acute/subacute youth SRC. Previous studies have also demonstrated diffusivity changes within the corpus callosum after mTBI, including increased FA in subjects with a history of concussion (Orr et al., 2016) and decreased MD at 24 h and 8 days post injury (Lancaster et al., 2016), in agreement with our observations. Other studies, however, have demonstrated diffusivity changes in the opposite direction, including decreased FA and increased RD in both symptomatic and recovered athletes (Churchill et al., 2018), decreased FA at 3 months post-injury (Wu et al., 2018), increased MD at 24–48 h after injury, 7 days after return-to-play, and 6 months after injury (Wu et al., 2020). Given the opposite direction and persistence of these findings even with recovery, it is likely that the mechanisms of injury differ from our subject group. Without additional forms of MRI, determination of possible mechanisms is limited.

One might expect there would be changes in functional connectivity of cortical regions associated with the symptoms impacted by injury to the corpus callosum; however, we did not observe functional connectivity changes for any of the cortical regions we investigated. It is possible that our ROIs did not directly correspond to the cortical regions impacted by the axonal injury of the corpus callosum, or that functional connectivity of our ROIs is more associated with specific symptoms within the SCAT assessment and not overall severity score. Further studies with larger symptom-specific subgroups could allow more in-depth assessment of cortical functional connectivity. We did, however, observe reduced connectivity between the caudate and each of the pallidum and thalamus in the recently concussed group, in addition to an acute day of MRI dependence in caudate CBF and pallidum ALFF. This is in agreement with a previous animal study of TBI reporting significant functional disturbances (injury-induced glucose hypometabolism) in areas including the caudate following repeated concussions, at eight days post first injury (Blaya et al., 2020). Further evidence of disruption within the basal ganglia and thalamocortical pathways was shown in a recent study that described local and large-scale beta oscillatory dysfunction in male mTBI subjects, indicating disrupted information flow through cortico-basal ganglia-thalamic circuits (Zhang et al., 2020). An fMRI study of working memory in youth SRC showed less activation of left thalamus and left caudate in concussed subjects compared to controls during non-verbal tasks (Keightley et al., 2014). Our observed basal ganglia changes may, of course, be independent of the diffusivity reductions we observed in the mid-to-posterior regions of the corpus callosum; however, the proximity of the corpus callosum to the caudate could suggest that axonal injury in the mid-to-posterior corpus callosum contributes to functional changes of the nearby basal ganglia. This is supported by the similarities in the observed trajectories of the resolution of these changes over time. The changes in the corpus callosum and basal ganglia did not exhibit an association with symptom severity, but rather a dependence on the day of MRI post-injury. As with the cerebellum connectivity changes, this could also indicate a symptom-independent mechanism of injury, a more objective indicator of symptoms, or injury mechanisms that take longer to resolve. Additional imaging timepoints between the acute and recovered phases would help to observe potential trajectories of recovery of the group differences that were independent of symptom or day of MRI.

No group differences or associations with total symptom severity or day of MRI were observed for any other white matter tract ROIs (internal capsule, external capsule, uncinate fasciculus, corona radiata, anterior thalamic radiation). Previous studies have reported a variety of DTI findings and directions of effect for the internal capsule (Cubon et al., 2011; Lancaster et al., 2016; Murugavel et al., 2014; Sasaki et al., 2014), external capsule (Lancaster et al., 2016; Churchill et al., 2018), corona radiata (Bazarian et al., 2012; Borich et al., 2013; Virji-Babul et al., 2013; Sasaki et al., 2014; Orr et al., 2016; Churchill et al., 2018), uncinate fasciculus (Wu et al., 2018), and thalamic radiations (Murugavel et al., 2014). The reason for the lack of consensus is unclear. A multimodal MRI and targeted ROI approach would potentially allow better elucidation of white matter injury mechanisms; the inclusion of gray matter ROIs to such investigations would aid in the interpretation of white matter injury mechanisms, as disruption in gray matter function could suggest specific types of white matter injury. This was the approach of the current study.

The recovered group exhibited no significant differences from healthy control participants for any MRI measures in any ROIs, suggesting that recovery is associated with resolution of the brain changes we observed in the recently concussed group. Although MRI findings across previous studies are mixed, a number of studies have also demonstrated MRI-based abnormalities that return to control level values with recovery from symptoms (Meier et al., 2015, 2020; Madhavan et al., 2019; D’Souza et al., 2020); however, there is little consistency in the observations across studies.

Several studies, however, observed persistent MRI findings or even new MRI findings after recovery of symptoms. A number of these studies reported altered cortical CBF in clinically recovered groups (Maugans et al., 2012; Wang et al., 2015, 2016; Barlow et al., 2017), but over a wide variety of regions across studies and little agreement in the direction of effect. Other studies reported persistent increased susceptibility (Koch et al., 2018) and reduced functional connectivity of the default mode network (Zhu et al., 2015). One multimodal MRI study combining volumetric analysis, diffusion imaging and CBF in participants with a history of concussion, reported chronic reduction in frontal lobe volume and blood flow as well as changes in white matter within posterior cortical regions (Churchill et al., 2017). This latter study is an example of how multimodal MRI can more comprehensively evaluate long-term effects of concussion. Given that our findings indicate that there are changes in MRI-based metrics of structure and function that are independent of symptoms, it is thus possible that persistent findings following recovery described in previous studies may be associated with symptoms not captured by assessment or they are adaptive responses of the brain to alleviate symptoms, thereby reducing the clinical significance of such findings. More study into the source of persistent MRI findings is warranted.

Our study had several limitations. While our participant groups were of similar age, sex and sport, despite our best efforts, we were not able to fully match them. Also, we were not able to collect MRI data from the recently concussed group after they had recovered, nor were we able to collect MRI data from the recovered group soon after injury. This places limits on conclusions regarding the resolution of MRI findings within the same participant; however, the similarity in initial concussion severity between groups increases confidence in our cross-sectional design. Our study was also potentially limited by low participant numbers, such that individual symptom-based analyses could not be performed. Moreover, the SCAT relies on self-reporting of symptoms, which can differ substantially between youth. Symptom assessment can be improved by combining multiple assessment tools or tools that incorporate parent input. With more participants and more comprehensive symptom assessment, the power of multimodal MRI for the investigation of specific post-concussive symptoms can be better exploited. In addition, while corrections for multiple comparisons were performed when investigating group means for the significant factors in our ANCOVAs, further adjustment of significance level was not implemented across modalities because means across modalities were not directly compared. With more participants and early MRI, between-modality models can be constructed to elucidate the relative contribution of each MRI modality to the description of concussion injury. Other demographic factors, such as subject race and socioeconomic status, were not considered in the current study; however, moving forward toward symptom-based analyses, these factors will become important and must be considered. Similar to any ROI based approach, while the current findings help to better elucidate the possible alterations in the selected ROIs, there could be other regions not included in this study by design that might be relevant to concussion. There also exist other forms of MRI that were not included in the present study, such as susceptibility-weighted imaging (SWI) for the assessment of microbleeds and fluid-attenuated inversion recovery (FLAIR) images to assess white matter hyperintensities. Our recent SWI study found no increase in microbleeds in youth mTBI participants with prolonged symptoms, relative to controls (Virani et al., 2021), and another group demonstrated no increase in FLAIR hyperintensities or SWI microbleeds in a SRC cohort, relative to controls (Jarrett et al., 2016). Thus, we chose not to include these forms of MRI in the current study.

In summary, we found that youth SRC is associated with acute cerebellar inflammation accompanied by reduced cerebellar activity and cerebellar-parietal connectivity, as well as structural changes of the mid-posterior regions of the corpus callosum accompanied by functional changes of the caudate, all of which resolve with recovery. While causality of these findings cannot be determined from the present study, our study demonstrates the need for a multimodal MRI assessment of brain structure and function to elucidate and interpret brain mechanisms of concussion injury and symptoms. Our findings also indicate that early MRI post-injury is important to exploit the potential for objective MRI-based indicators for concussion diagnosis, recovery assessment and prediction of outcome.

## Data availability statement

The raw data supporting the conclusions of this article will be made available by the authors, without undue reservation.

## Ethics statement

The studies involving human participants were reviewed and approved by University of Calgary Conjoint Health and Research Ethics Board. Written informed consent to participate in this study was provided by the participants’ legal guardian/next of kin.

## Author contributions

NP: conceptualization, methodology, formal analysis, investigation, writing—original draft, and visualization. CD: writing—review and editing, data curation, and funding acquisition. SD, AH, and KY: writing—review and editing. BB: resources. CE: conceptualization, formal analysis, investigation, writing—review and editing, validation, resources, supervision, project administration, and funding acquisition. BG: conceptualization, methodology, formal analysis, investigation, writing—original draft, visualization, validation, resources, supervision, and project administration. All authors contributed to the article and approved the submitted version.

## Footnotes

1
https://fsl.fmrib.ox.ac.uk/fsl/fslwiki

2
https://surfer.nmr.mgh.harvard.edu

3
https://fsl.fmrib.ox.ac.uk/fsl/fslwiki/eddy

4
https://fsl.fmrib.ox.ac.uk/fsl/fslwiki/FDT

5
https://fsl.fmrib.ox.ac.uk/fsl/fslwiki/TBSS

6
https://fsl.fmrib.ox.ac.uk/fsl/fslwiki/MELODIC

7
http://rfmri.org/dpabi
